# Superconducting edge states in a topological insulator

**DOI:** 10.1038/s41598-021-97558-z

**Published:** 2021-09-15

**Authors:** I. V. Yurkevich, V. Kagalovsky

**Affiliations:** 1 School of Informatics and Digital Engineering, Aston University, Birmingham, B4 7ET UK; 2 Shamoon College of Engineering, Bialik/Basel St., 84100 Beer-Sheva, Israel

**Keywords:** Topological matter, Topological insulators

## Abstract

We study the stability of multiple conducting edge states in a topological insulator against perturbations allowed by the time-reversal symmetry. A system is modeled as a multi-channel Luttinger liquid, with the number of channels equal to the number of Kramers doublets at the edge. Assuming strong interactions and weak disorder, we first formulate a low-energy effective theory for a clean translation invariant system and then include the disorder terms allowed by the time-reversal symmetry. In a clean system with *N* Kramers doublets, *N* − 1 edge states are gapped by Josephson couplings and the single remaining gapless mode describes collective motion of Cooper pairs synchronous across the channels. Disorder perturbation in this regime, allowed by the time reversal symmetry is a simultaneous backscattering of particles in all *N* channels. Its relevance depends strongly on the parity if the number of channel *N* is not very large. Our main result is that disorder becomes irrelevant with the increase of the number of edge modes leading to the stability of the edge states superconducting regime even for repulsive interactions.

## Introduction

Topological insulators (TI) have been a subject of intensive research in condensed matter physics^1,2^. Each conducting edge state in TI is a helical Kramers doublet (KD) with opposite spins propagating in opposite directions. Time-reversal symmetry (TRS) can protect the existence of conducting edge states, since it forbids a spin-flip backscattering within the same KD, but allows it between two different KDs. If there is an even number of KDs in a non-interacting system then a backscattering between different doublets generated by a disorder localises all edge states and the system is a trivial insulator. On the other hand, at least one channel remains delocalised if the number of KDs is odd^3^ and the system is a topological insulator. Thus the parity of the number of KDs defines whether the insulator is topological or trivial. The symmetry of the scattering matrix^3^ used to prove this conclusion is valid for non-interacting systems only. The robustness of edge states in the presence of interactions was studied intensively for a system with a single KD^4–7^, and for systems with one or more KDs^8–12^. It was found that an even number of KDs can be stabilised by interactions and remain conducting. The existing experiments discovered so far a 2D TI with a single KD^13^ only.

In our previous study^14^ we have shown that in the clean system with *N* Kramers doublets there *always* exist *N* − 1 relevant perturbations (either of superconducting or charge density wave character), which *always* open *N* − 1 gaps. We have then investigated in detail the effect of disorder in the charge density wave regime, and showed that the interacting system with *N* Kramers doublets at the edge may be either a trivial insulator or a topological insulator for *N* = 1 or 2, whereas any higher number $N>2$ of doublets gets fully localized by disorder pinning, irrespective of the parity issue.

### Model

In this paper, we consider the effect of disorder in the superconducting (SC) regime, when Josephson coupling is relevant in a clean system. We start with a brief description of a multichannel Luttinger liquid constructed to study a topological insulator with *N* edge states^14^. Two vector fields, ${{\boldsymbol{\phi }}}^{\rm T}=(\phi _1\,,\ldots \,,\phi _N)$ and ${{\boldsymbol{\theta }}}^{\rm T}=(\theta _1\,,\ldots \,,\theta _N)$, parametrising excitation densities, $\rho _i=\partial _x\phi _i/2\pi$, and currents, $j_i=\partial _x\theta _i/2\pi$, in each channel *i* ($1\le i \le N$) are introduced^15–20^. The Lagrangian, ${{\mathscr {L}}}_0$,

1 $$\begin{aligned} {{\mathscr {L}}}_0=\frac{1}{8\pi }({{\boldsymbol{\phi }}}^{\rm T},\,{{\boldsymbol{\theta }}}^{\rm T})\,\left[ {{\hat{\tau }}}_1\,\partial _t+{{\hat{V}}}\,\partial _x\right] \,\partial _x\,\left(\begin{array}{l} {{\boldsymbol{\phi }}}\\ {{\boldsymbol{\theta }}} \end{array}\right), \end{aligned}$$

includes block-diagonal matrix ${{\hat{V}}}={\rm diag}[{{\hat{V}}}_+\,,{{\hat{V}}}_-]$ with each block describing density-density, ${{\hat{V}}}_+$, and current-current, ${{\hat{V}}}_-$, interactions; ${{\hat{\tau }}}_1$ is the Pauli matrix. We follow^14^, and consider identical interactions between all the channels

2 $$\begin{aligned} V^{ij}_{\pm }=\left( 1+g_{\pm }\right) \,\delta _{ij}+g'_{\pm }\,\left( 1-\delta _{ij}\right) \,, \end{aligned}$$

All parameters are defined following standard nomenclature: $g_{\pm }=g_4\pm g_2$ with coupling $g_4$ being an interaction strength between electrons moving in the same direction (right- with right-movers, and left- with left-movers), and $g_2$ is the interaction strength between electrons moving in the opposite directions within the same KD. The couplings with prime have similar meaning for inter-channel interactions.

Introducing two projectors, ${{\hat{\Pi }}}_{\perp }$ and ${{\hat{\Pi }}}_{\parallel }={\hat{\mathbbm{1}}}-{{\hat{\Pi }}}_{\perp }$,

3 $$\begin{aligned} {{\hat{\Pi }}}_{\perp }=N^{-1}\,{{\varvec{e}}}\otimes {{\varvec{e}}}^{\rm T}\,,\quad {{\varvec{e}}}^{\rm T}=\left( 1\,\ldots , 1\right) \,, \end{aligned}$$

one can write both interaction matrices ${{\hat{V}}}_{\pm }$ as a linear combination of them:

4 $$\begin{aligned} {{\hat{V}}}_{\pm }={{\hat{V}}}_{\pm }^{\parallel }{{\hat{\Pi }}}_{\parallel }+{{\hat{V}}}_{\pm }^{\perp }{{\hat{\Pi }}}_{\perp }\,, \end{aligned}$$

where

5 $$\begin{aligned} V^{\parallel }_{\pm }= 1+g_{\pm }-g'_{\pm }\,, \end{aligned}$$

6 $$\begin{aligned} V^{\perp }_{\pm }= 1+g_{\pm }+(N-1)\,g'_{\pm }\,. \end{aligned}$$

It is known that RG flow for a single channel problem depends on a single parameter (so-called Luttinger parameter) $K=\sqrt{(1+g_-)/(1+g_+)}$ while excitations velocity does not play any role in the renormalisation. The general construction^21^ generalises this result: *N*-channel Luttinger liquid is described by *N* velocities and a real symmetric $N \times N$ matrix which we call Luttinger ${\hat{K}}$-matrix responsible for the impurity strength renormalisation^21–26^. This matrix must be found from the algebraic matrix equation:

7 $$\begin{aligned} {{\hat{K}}}\,{{\hat{V}}}_{+}\,{{\hat{K}}}={{\hat{V}}}_{-}\,. \end{aligned}$$

It is this ‘Luttinger’ ${\hat{K}}$-matrix that defines the scaling dimensions of all possible scattering terms in all possible phases. Solving this equation for the interaction matrices ${{\hat{V}}}_{\pm }$ in Eq. (4), one finds:

8 $$\begin{aligned} {{\hat{K}}}=K_{\parallel }{{\hat{\Pi }}}_{\parallel }+K_{\perp }{{\hat{\Pi }}}_{\perp }\,, \end{aligned}$$

where

9 $$\begin{aligned} K_{\parallel }=K\,\sqrt{\frac{1+(N-1)\alpha _-}{1+(N-1)\alpha _+}}\,,\quad K_{\perp }=K\,\sqrt{\frac{1-\alpha _-}{1-\alpha _+}}\,, \end{aligned}$$

and $$\alpha _{\pm }=g'_{\pm }/(1+g_{\pm })$$.

### Anharmonic interaction terms

The most general interaction (beyond forward-scattering quadratic terms in the Lagrangian) can be written as

10 $$\begin{aligned} {{\mathscr {L}}}_{\rm int}=\sum \limits _{Q=0}\,h(\mathbf{j},\mathbf{q})\,\exp \left[ i(\mathbf{j}{{\boldsymbol{\phi }}}+\mathbf{q}{{\boldsymbol{\theta }}})\right] \,, \end{aligned}$$

where vectors $\mathbf{j}$ and $\mathbf{q}$ have components $j_i$ and $q_i$ that either both integer or half-integer. The vertices $\exp \left[ i\mathbf{j}{{\boldsymbol{\phi }}}\right]$ are neutral while vertices $\exp \left[ i\mathbf{q}{{\boldsymbol{\theta }}}\right]$ carry charge $Q=2\mathbf{q}\mathbf{e}=2\sum _i\,q_i$. The summation is restricted by the neutrality requirement *Q* = 0 meaning that each term in the Hamiltonian conserves number of particles.

## Methods

Every perturbation in Luttinger liquid leads to correction to observables that scales with the temperature as a power law. The exponent of the power law is equal to the difference between the scaling dimension (dependent on the interaction parameters of the system and the perturbation) and the physical dimension *d* = 2 (one spatial and one temporal dimensions). If scaling dimension is higher than the physical one, the perturbation generates corrections which are small perturbations at low temperatures and vanish in zero-*T* limit. Such perturbations are irrelevant. If a perturbation has scaling dimension which is lower than *d* = 2, the ’correction’ becomes important at low temperatures and cannot be treated as a small correction because its divergent low-*T* behaviour wins over a small bare value of the coupling strength. Such perturbation is called relevant because it cannot be treated within a perturbation theory and instead it should be taking into account at the initial stage of formulating effective low-energy model describing low-temperature phases of the system.

To formulate effective low-energy model, we have to analyse scaling dimensions of various perturbations Eq. (10) to separate gapless and gapped degrees of freedom which correspond to irrelevant and relevant perturbations accordingly. The scaling dimension, $\Delta (\mathbf{j},\mathbf{q})$, of a vertex $e^{i(\mathbf{j}{{\boldsymbol{\phi }}}+\mathbf{q}{{\boldsymbol{\theta }}})}$ in the perturbation, ${{\mathscr {L}}}_{int}$, to the quadratic Lagrangian ${{\mathscr {L}}}_0$, is known^21–26^ to be

11 $$\begin{aligned} \Delta (\mathbf{j},\mathbf{q})={{\varvec{j}}}\cdot {{\hat{K}}}{{\varvec{j}}}+{{\varvec{q}}}\cdot {{\hat{K}}}^{-1}{{\varvec{q}}}\,. \end{aligned}$$

The explicit form of the *K*-matrix, Eq. (8), and the neutrality condition allow simplification,

12 $$\begin{aligned} \Delta (\mathbf{j}, \mathbf{q})=\frac{K_{\parallel }}{N}\,J^2+K_{\perp }{{\varvec{j}}}_{\perp }^2+K^{-1}_{\perp }{{\varvec{q}}}^2\,, \end{aligned}$$

where

13 $$\begin{aligned} J=\mathbf{j} \mathbf{e}\,,\qquad {{\varvec{j}}}_{\perp }={{\varvec{j}}}-(J/N){{\varvec{e}}}\,. \end{aligned}$$

Here *J* is the change of total momentum of particles involved in the process described by ($\mathbf{j}$, **q**)-term in anharmonic part of interactions, Eq. (10). Note that the neutrality requirement *Q* = 0 implies that *J* is an integer, while in a translation invariant system *J* = 0 reflecting the absence of scatterings not respecting the total momentum conservation.

It is necessary to stress the difference between this model of a topological insulator and the coupled wires constructions. The main distinction is the absence of single-particle inter-channel scattering terms amongst translation invariant *J* = 0 contributions in Eq. (10). All single-particle processes have been taking into account at the level of derivation of multi-channel Luttinger liquid where channels are eigenmodes of a single-particle two-dimensional Hamiltonian. These modes are orthogonal to each other and localised in a narrow region in the boundary layer of the two-dimensional sheet so that we may assume that density-density interactions are long-ranged on the scale of the modes separation. This fact justifies our model interaction Eq. (2).

### Most relevant perturbations

The possible amplitudes of the couplings are related to each other by hermiticity ${{\bar{h}}}(\mathbf{j},\mathbf{q})=h(-\mathbf{j},-\mathbf{q})$ and time-reversal symmetry (TRS)^11,14^:

14 $$\begin{aligned} h(\mathbf{j},\mathbf{q})=h(\mathbf{j},-\mathbf{q})\,(-1)^J\,. \end{aligned}$$

The most relevant inter-channel interactions in a clean system are the charge density wave (CDW) and superconducting (Josephson) couplings^14,20,27–32^. Assuming that interactions are strong and disorder is weak, we must first consider a translation-invariant (clean) system, imposing the restriction *J* = 0 (momentum conservation), and work out the low-energy Hamiltonian keeping only gapless modes, and only then add the disorder terms allowed by symmetries^33^. The scaling dimensions, Eq. (12), is a quadratic function of the lengths of vectors ${{\varvec{j}}}$ and ${{\varvec{q}}}$ and, therefore, the most dangerous perturbations correspond to the shortest vectors allowed by the symmetries. These perturbations are CDW,

15 $$\begin{aligned} {{\mathscr {L}}}^{\rm cdw}\sim \sum _{i,j}\,e^{i(\phi _i-\phi _j)}\,, \end{aligned}$$

and SC couplings,

16 $$\begin{aligned} {{\mathscr {L}}}^{\rm sc}\sim \sum _{i,j}\,e^{i(\theta _i-\theta _j)}\,. \end{aligned}$$

In a clean system, the scaling dimensions are known to be $\Delta ^{\rm cdw}=2K_{\perp }$ and $\Delta ^{\rm sc}=2K^{-1}_{\perp }$^14^. We will focus on the situation when the Josephson-type superconducting interaction is relevant, i.e. $K_{\perp } > 1$. It is important to note that this condition does not necessarily mean attraction between electrons. Strong enough inter-channel repulsion $0< \alpha _+ < 1$ may lead to this situation, see Eq. (9).

In the absence of single-particle inter-mode tunnelling, the Josephson coupling ${{\mathscr {L}}}^{\rm sc}$ stems from the inter-channel Coulomb interactions written in a basis of the single-particle eigenstates (edge modes). If a single-particle two-dimensional Hamiltonian with spin-orbit interaction is projected onto low-energy right- and left-moving modes existing near the boundary and described by the wavefunctions $R_i(x,y)$ and $L_j(x,y)$, the terms like in Eq. (16) are generated by the Coulomb interaction projected onto the space of the edge modes. The effective Hamiltonian will contain terms in Eq. (16) with

17 $$\begin{aligned} {{\mathscr {L}}}^{\rm sc}=\sum _{i,j}\,h^{\rm sc}_{ij}\,e^{i(\theta _i-\theta _j)}\,, \end{aligned}$$

where

18 $$\begin{aligned} h^{\rm sc}_{ij}=-\langle R_i, L_j|U|L_j, R_i\rangle \,. \end{aligned}$$

Assuming that interaction $U(|{{\varvec{r}}}-{{\varvec{r}}}'|)$ is smooth on the scale of the edge mode decays in the transverse to edge *y*-direction, we may restrict our consideration to a collective Josephson coupling:

19 $$\begin{aligned} {{\mathscr {L}}}^{\rm sc}\rightarrow h^{\rm sc}\sum _{i,j}\,e^{i(\theta _i-\theta _j)}\,. \end{aligned}$$

The sign of the Josephson coupling constant in this scenario is not determined by the sign of the Coulomb interaction *U* but rather by the matrix elements in Eq. (18). We assume a positive sign that corresponds to the alignment of superconducting $\theta$-phases. The situation when *h* is negative and leads to a frustration of the superconducting phases will be considered elsewhere.

The inter-channel charge density wave terms generate phase-slip process that tends to destroy superconductivity^34,35^. We will neglect this mechanism because it is irrelevant in the renormalisation group (RG) sense at $K_{\perp } > 1$^14^.

In this paper, our focus will be on the regime $K_{\perp } > 1$ where the inter-channel SC perturbations, corresponding to the tunnelling of Cooper pairs between channels is a relevant perturbation. Unlike the wire construction of multi-channel Luttinger liquids, there is no single-particle tunnelling between edge modes that are eigenstates of the non-interacting Hamiltonian. The most relevant inter-channel superconducting couplings are the Josephson-type terms.

The superconducting state, when all terms in ${{\mathscr {L}}}^{\rm SC}$ Eq. (16) are relevant, will be sensitive to the signs of the Josephson couplings $h^{\rm sc}_{ij}$. We will assume that all Josephson couplings $h^{\rm sc}_{ij}$ are negative because it is natural to expect that the tunnelling of the Cooper pairs between channels must lead to the superconducting phase homogeneity across the channels $\theta _i=\Theta /\sqrt{N}$ for $i=1,\ldots , N$. This collective (‘centre-of-mass’) superconducting phase and its conjugate field $\Phi$ are described by the effective low-energy Lagrangian,

20 $$\begin{aligned} {{\mathscr {L}}}_{\parallel }=\frac{1}{4\pi }\,\partial _t\Theta \,\partial _x\Phi - \frac{v_{\parallel }}{8\pi }\left[ \frac{1}{K_{\parallel }}\left( \partial _x\Phi \right) ^2 +K_{\parallel }\left( \partial _x\Theta \right) ^2\right] \,. \end{aligned}$$

### Haldane criterion

Now we can study the effect of disorder on the remaining conducting channel. The disorder breaks momentum conservation and we must include all terms with $J\ne 0$ allowed by the symmetry. There is another constraint on the type of terms that we are allowed to add, they must be compatible with the condition that (*N* − 1) modes have been frozen, i.e. the new added terms must commute with those we used to decide which modes are gapped. This criterion was formulated by Haldane^36^. In our notations, the Haldane criterion can be written as

21 $$\begin{aligned} \mathbf{j}\cdot \mathbf{q}'=\mathbf{j}'\cdot \mathbf{q}\,, \end{aligned}$$

where $(\mathbf{j},\mathbf{q})$ are vectors characterising the relevant perturbations which have been taking into account when analysing the clean system and led to (*N* − 1) modes being gapped, while $(\mathbf{j}', \mathbf{q}')$ are the vectors of the allowed perturbations. This criterion imposes different conditions on allowed disorder perturbations for different regimes.

In the SC regime, that we study here, the clean system was perturbed by the Josephson interaction terms with vectors $\mathbf{j}=0$, and $\mathbf{q}=(0,\ldots ,1_i, \ldots , -1_j, \ldots , 0)$. Due to the neutrality condition, the perturbation term will not contain the field $\Theta$ because the corresponding exponential will be $\mathbf{q}'\cdot \mathbf{e}\,\Theta =0$, effectively meaning $\mathbf{q}'=0$. The second vector in the possible allowed perturbation $\mathbf{j}'$ has to be orthogonal to $\mathbf{q}$ according to the Haldane criterion Eq. (21), but $\mathbf{q}$ is always orthogonal to $\mathbf{e}$ because of the neutrality. We therefore conclude that the additional allowed disorder perturbation in the SC regime should be parallel to the vector $\mathbf{e}$, and since both components of vectors $\mathbf{q}'=0$ and $\mathbf{j}'$ must have the same parity, we conclude that $\mathbf{j}'=n\mathbf{e}$, $\mathbf{q}'=0$, where *n* is an integer:

22 $$\begin{aligned} {{\mathscr {L}}}_{dis}=D\,e^{in\,{{\varvec{e}}}{{\boldsymbol{\phi }}}}+\mathrm{c. c.} \end{aligned}$$

The TRS requirement, Eq. (14), implies that $J'=\mathbf{j}'\cdot \mathbf{e}=nN$ is an even number. We may keep only the most relevant terms which correspond to the minimal allowed values of *n*. This means that $J'=N$ (describing simultaneous back-scattering of *N* particles in all *N* channels) for even number of channels, and $J'=2N$ (simultaneous back-scattering of 2*N* particles) for odd number of channels. The scaling dimension of the disorder perturbation Eq. (22) is obtained from Eq. (12) with $J'=pN$ where parity parameter *p* differentiates odd (*p* = 2) and even (*p* = 1) number of channels:

23 $$\begin{aligned} \Delta _{J'}=J'^2K_{\parallel }/N=p^2K_{\parallel }N\,, \end{aligned}$$

differs significantly.

Now we present a phase digram illustrating the stability of the SC regime. We follow^14^ and consider only density-density interactions, i.e. assume only current–current interaction matrix $\hat{V}_-=\hat{\mathbbm{1}}$ in Eq. (2). The two parameters *K* and $\alpha _+$ characterise intra- and inter-mode interactions, respectively, and define the effective Luttinger parameters,

24 $$\begin{aligned} K_{\perp }= K\,(1-\alpha _+)^{-1/2}\,, \end{aligned}$$

25 $$\begin{aligned} K_{\parallel }= K\,\left[ 1+(N-1)\alpha _+\right] ^{-1/2}\,. \end{aligned}$$

We will focus now on the *repulsive density–density interaction*, ($0<K<1$ and $0<\alpha _+<1$), and demonstrate that, nevertheless, the SC regime is possible. A conducting mode is robust in a SC regime if disorder is irrelevant. This region of existence is defined by disorder irrelevance, $\Delta _{J'} > 3/2$, and depends on the parity (*p* = 1 and *p* = 2 for even and odd channel number correspondingly):

26 $$\begin{aligned} (N-1)\,\alpha _+ < \left( \frac{2K}{3}\right) ^2N^2\,p^4-1\,. \end{aligned}$$

**Figure 1:**
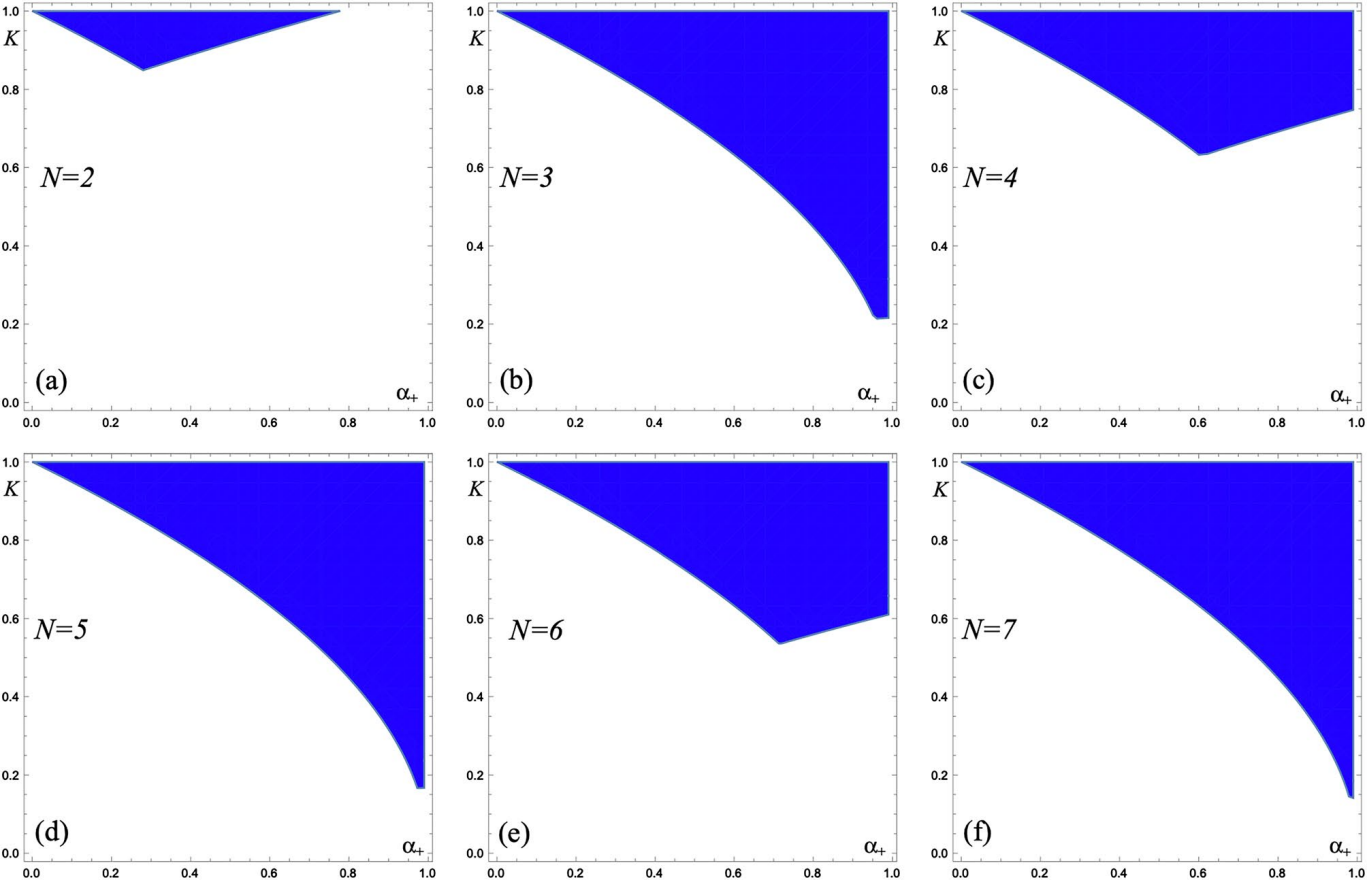
The phase diagram for a set of *N* Kramers doublets under repulsive density–density interaction. The blue regions represent stable SC regime.

We immediately observe that the disorder is much less relevant (large scaling dimension) for odd number of channels than for even as long as a number of channels *N* is not too large. As *N* is increasing, this difference disappears and only SC regime condition defines the stability of the mode, independent on parity of the number of channels. Figure 1 illustrates these properties. Figure 1a shows a stability (blue) region for *N* = 2, which is much smaller than stability region for *N* = 3 in Fig. 1b. If we increase *N*, stability regions for systems with even number of channels increase significantly (see Fig. 1c for *N* = 4 and Fig. 1e for *N* = 6), whereas for odd *N* the increase is very small (see Fig. 1d for *N* = 5). For $N\ge 7$ (*N* = 7 stability region is shown in Fig. 1f) the stability region practically saturates (is defined only by SC regime condition, $K_{\perp }>1$, independent on the parity of *N*.

As one can see from the phase diagrams in Fig. 1, inter-mode repulsion ($0\le \alpha _+\le 1$) initially drives the system into a superconducting state through the enhancement of inter-mode Josephson coupling. Further increase of the inter-mode repulsion enhances effect of disorder which would finally suppress superconducting correlations but the relevance of disorder is very sensitive to the parity of mode number. The time reversal symmetry requires twice as many particles to be backscattered for odd parity as compared to the even one. This leads to a relatively weak effect of disorder onto systems with odd number of modes. This distinction is obvious in the phase diagrams in Fig. 1.

It is necessary to stress that superconducting regions in the phase diagrams Fig. 1 correspond to the power-law decay of both density–density ($\sim x^{-2}$) and Cooper pairing ($\sim x^{-2/K_{\parallel }}$) correlation functions. Although superconducting correlations decay faster than the density ones (since $K_{\parallel } < 1$), we call these regions superconducting because superconducting correlations there cannot be destroyed by disorder contrary to what happens in weakly interacting regimes.

The results presented in this paper together with our recent results^14^ conclusively show that one of the two two-particle interactions always becomes relevant and the system, therefore, enters either CDW or SC regime. This transition takes place with decreasing temperature when the renormalised dimensionless perturbation amplitude reaches the order of unity. Different temperature dependencies of the amplitudes, $h^{CDW}\sim T^{2K_{\perp }-2}$ and $h^{SC}\sim T^{2K_{\perp }^{-1}-2}$ can be studied experimentally as in^13^ but at lower temperatures. Such experiments could confirm the destruction of the topological insulator by disorder or its robustness depending on interaction parameters and the number of edge states.

The predicted in this paper edge superconductivity should be also measurable in experimental setups similar to those designed to analyse surface superconductivity in three-dimensional samples (see, for example^37^, and the references there). The measurements carried out with a SQUID magnetometer must record dependence of a magnetic moment of the sample in a weak magnetic field. The edge current will not be able to expel the flux but it has to be observed and scale with temperature as a power law with non-universal, system dependent, exponent which is a distinct feature of the Luttinger liquid physics.

## Conclusions

We have studied a topological insulator with *N* Kramers doublets at the edge in the model of long range featureless interaction. We have shown that when a system is in the superconducting regime, the Josephson couplings open (*N* − 1) gaps and the disorder affects the only remaining centre-of-mass mode but, nevertheless, its effect depends on the parity the system had in the normal state. The scaling dimension and, therefore, the phase diagram are sensitive to the parity of the number of channels *N* for few channel case, and parity effect disappears for $N\ge 7$. We wish to stress that due to interchannel coupling even repulsive interactions may lead to a regime with dominant superconducting correlations.

## Data Availability

The data that support the findings of this study are available from the corresponding authors upon reasonable request.
